# An Integrated Architecture for Colorectal Polyp Segmentation: The µ-Net Framework with Explainable AI

**DOI:** 10.3390/diagnostics15222890

**Published:** 2025-11-14

**Authors:** Mehedi Hasan Emon, Proloy Kumar Mondal, Md Ariful Islam Mozumder, Hee Cheol Kim, Maria Lapina, Mikhail Babenko, Mohammed Saleh Ali Muthanna

**Affiliations:** 1 Institute of Digital Anti-Aging Healthcare, Inje University, Gimhae-Si 50834, Republic of Korea; mehedihasanemon3913@gmail.com (M.H.E.); proloykumar1996@gmail.com (P.K.M.); arifulislamro@gmail.com (M.A.I.M.); heeki@inje.ac.kr (H.C.K.); 2 Next Station AI, Research & Development Center, Dhaka 1216, Bangladesh; 3 Research Center for Trusted Artificial Intelligence, Ivannikov Institute for System Programming of the Russian Academy of Science, 109004 Moscow, Russia; 4 Department of Computational Mathematics and Cybernetics, North-Caucasus Federal University, 355017 Stavropol, Russia; mgbabenko@ncfu.ru; 5 Faculty of Computing and IT, Sohar University, Sohar 311, Oman; muthanna@sfedu.ru; 6 Department of International Business Management, Tashkent State University of Economics, Tashkent 100066, Uzbekistan

**Keywords:** µ-Net, colorectal cancer, polyp segmentation, deep learning, explainable artificial intelligence

## Abstract

**Objectives:** Colorectal cancer (CRC) is the second-deadliest cancer globally, with an estimated 52,900 additional deaths expected in the United States by 2025. Early detection through colonoscopy significantly reduces CRC mortality by enabling the removal of pre-cancerous polyps. However, manual visual inspection of colonoscopy images is time-consuming, tedious, and prone to human error. This study aims to develop an automated and reliable polyp segmentation and classification method to improve CRC screening. **Methods:** We propose a novel deep learning architecture called µ-Net for accurate polyp segmentation in colonoscopy images. The model was trained and evaluated using the Kvasir-SEG dataset. To ensure transparency and reliability, we incorporated Explainable AI (XAI) techniques, including saliency maps and Grad-CAM, to highlight regions of interest and interpret the model’s decision-making process. **Results:** The µ-Net model achieved a Dice coefficient of 94.02%, outperforming other available segmentation models in accuracy, indicating its strong potential for clinical deployment. Integrating XAI provided meaningful visual explanations, enhancing trust in model predictions. **Conclusions:** The proposed µ-Net framework significantly improves the Precision and efficiency of automated polyp screening. Its ability to segment, classify, and interpret colonoscopy images enables early detection and supports clinical decision-making. This comprehensive approach offers a valuable tool for CRC prevention, ultimately contributing to better patient outcomes.

## 1. Introduction

**Figure 1:**
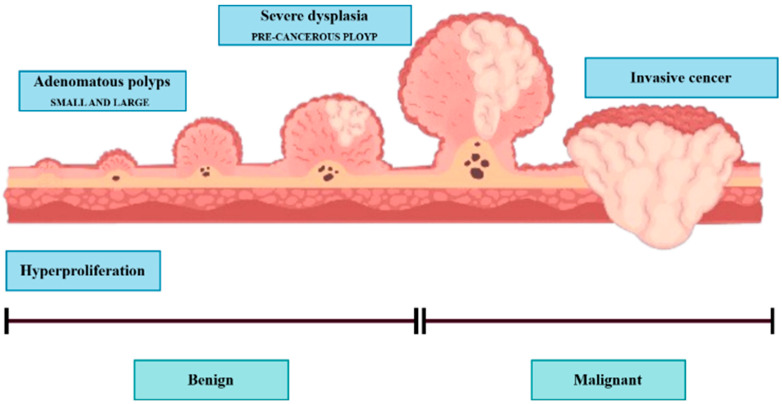
Polyps are generally divided into three stages: (1) hyperplastic stage, (2) adenomatous or precancerous stage, (3) malignant or CRC stage.

Colonoscopy is the preferred method to identify colorectal lesions. Colorectal lesions can be timely recognized and removed during a colonoscopy examination, and polyps can be detected at an early stage to prevent disease progression. The localization of polyps in medical image segmentation (PS) tasks is an early-stage challenge with the potential to significantly contribute to the clinical prevention of colorectal cancer. The digestive system comprises the gastrointestinal (GI) tract and other vital organs, including the liver, gall bladder, and pancreas. The GI tract is not part of the digestive system but is rather a part of the passageway that comes into the digestive system. The GI tract is composed of a series of hollow organs that food, liquids, and digestible products pass through, including the mouth, throat, esophagus, stomach, small intestine (comprising the duodenum, jejunum, and ileum), large intestine, or colon, and rectum. The colon (large intestine) may also be primarily involved with infections, bacteria, viruses, or parasites, causing mucosal conditions such as ulcers and polyps. Some polyps may become cancerous and can cause colorectal cancer (CRC), which can potentially be life-threatening to the patient. However, when found early, they can be successfully treated, and the risk of CRC decreases. Polyps are growths from the surface of the mucus, which grows on the body’s surface. They often resemble tiny lumps or small mushrooms. While polyps mostly grow in the colon, they can also occur in the uterus, ear canal, cervix, stomach, nose, and throat. Some polyps are not pre-cancerous or benign; others can transform into cancer and grow lines, as shown in Figure 1.

Hyperplastic polyps are generally tiny and located at the end of the colon, like the rectum or sigmoid colon. They are typically less likely to be malignant. Approximately two-thirds of colon polyps are adenomatous polyps, some of which can develop into cancer. Polyps are also graded by size, overall appearance, and what is revealed when looking at them under the microscope. The bigger the polyp is, the more chances there are of it converting into cancer. For this reason, urologists suggest the removal of polyps larger than 5 mm. Polyp cells of the adenomatous stage have the potential to evolve to malignant CRC and then to invasive CRC, a grave risk. Most polyps are asymptomatic [1,2], and the optimum means of preventing CRC is not to avoid polyps but to monitor and screen the colon and rectum. Thus, pre-cancerous polyps can be screened and detected by colonoscopy. However, the distinction between hyperplastic polyps and adenomatous polyps by colonoscopy vision is not convenient, and urologists have determined the results of colon biopsies in most cases. By virtue of being an endoscopy, and even though its main role is the observation of the entire digestive tract, including the esophagus, stomach, duodenal portion of the small intestine, colon, and rectum, it was recognized and confirmed that colonoscopy is the best method to visualize the entire digestive tract from inside. These include gastroscopy (upper endoscopy) and colonoscopy (lower endoscopy). Colonoscopy allows a doctor to look inside a person’s colon using a long, flexible tube with a light and a camera at one end, called an endoscope, to look at the entire colon. The endoscope contains a light and a small camera that allows it to see an organ without major cuts. For a colorectal examination, you might have a colonoscopy to examine the rectum, colon, and large intestine. The analysis is conducted by hand in current practice using a naked eye visual assessment by a skilled gastroenterologist. Nevertheless, this is a laborious, fatigue-prone procedure with inter- and intra-observer variation. Also, the expert-dependent tests retard evidence-based health care in a world short of health care workers [3], and are another instance of automatic inspection for effective, objective, and precise colonoscopy. Here we present a method of automatic colonoscopy examination with a polyp segmentation, which can distinguish polyps from colonoscopy images. In most colonoscopy inspections, the diagnosis of irregular lesions is performed by an expert, and this process is completely dependent on his expertise and knowledge, which may result in missed crypts. According to Borgoli et al. [4], this comes at a time when identification of polyps is known to be suboptimal in colonoscopy; 20% of cases are misidentified. Automatic polyp segmentation can aid practitioners by automatically segmenting polyps extracted from colonoscopy images or videos. This automation has drawn the attention of computer vision specialists, and hence, numerous research studies have been dedicated to autonomous polyp detection. However, such methods are not yet accurate enough. Another issue is that methods proposed by different groups were trained and tested on various datasets, complicating the comparison. In 2020, Hicks et al. released the Endotect Challenge dataset [5] to allow comparison with polyp detection methods and trained the segmentation network for the proposed system using the Hyper-CVS dataset. He tested it using two standard databases, the Endotect [5] Challenge Test Image and the CVS-SEG [6] databases. Polyp morphological classification and risk assessment are inseparable parts of colonoscopy screening. Computer-assisted methods are restricted to polyp segmentation only, and no degree of polyps has been analyzed. Polyp segmentation and classification should be part of an autonomous colonoscopy examination system.

Pointwise Contribution:

• We analyze various segmentation-based model architectures and observe some potential improvements.
• Considering the need for development, we proposed µ-Net, a strongly improved version of the traditional U-Net model.
• We experimented with CRC polyp image datasets obtained via different modalities; µ-Net exhibited superior performance.
• We qualitatively examined some challenging images and observed significant improvements when using µ-Net rather than U-Net.

The rest of this article is as follows: Section 2 provides an overview of related work. Section 3 explains the experimental details. Section 4 introduces this paper’s experiment. Explainable AI is supposed to explain AI results (see Section 5), and the experimental results are presented in Section 5. Section 6 offers a discussion, and finally, we conclude our research work.

## 2. Related Work

Effective Convolutional Neural Networks (CNNs) and Transformers are two of the most influential architectures in deep learning. They have revolutionized their respective domains, image segmentation and classification, and are at the forefront of AI research. Below, we discuss the development, applications, and advancements in CNNs and transformers, highlighting key studies and research trends.

### 2.1. Convolutional Neural Networks

**Figure 2:**
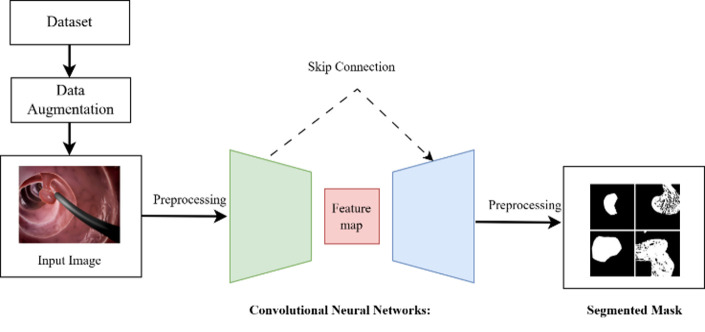
A CNN workflow for segmentation.

Convolutional Neural Networks (CNNs) and Transformers are architectures with the most significant influence in deep-learning. They have revolutionized their domains—image segmentation and classification—and remain at the forefront of AI research. Below, we discuss the development, applications, and advancements in CNNs and transformers, highlighting key studies and research trends. Figure 2 shows a CNN workflow for segmentation. Computerized polyp segmentation is significant in clinics for decreasing cancer-related death rates. CNNs are the standard choices for medical image segmentation, and many of the well-known architectures for 2D image segmentation were employed for this task. (a): U-Net [7], an encoder–decoder model first proposed for biomedical image segmentation. The structure of the U-Net is relatively simple and effective, and it performs well on several medical image segmentation. Nevertheless, it may not work well for complicated or diversified input images, and one can choose the alternative methods. PraNet [8] is a CNN tailored for automatic polyp segmentation in colonoscopy images. It applies a parallel partial decoder, extracts high-level structural information from the photos, and forms a global map for additional processing. Meanwhile, an inverse attention module is employed for boundary signal mining, aiding in capturing cross-region relations in the image. To address the issue of misaligned predictions and enhance the segmentation performance, PraNet leverages an iterative cooperation mechanism.

Experiments show that PraNet achieves state-of-the-art results on various image segmentation benchmarks with real-time performance at around 50 FPS. DeepLabV3+ [9] is a state-of-the-art network for semantic image segmentation based on atrous convolution, providing better contextual dependencies and feature extraction at multiple scales. This approach allows more accurate segmentation of objects with complex shapes or large variations in scale, but is computationally more expensive, and training/estimation can be slow. HRNetV2 [10,11] is a CNN for human pose estimation that can transfer multi-scale information between the layers with (or without) different resolutions. This network may do better on small or fuzzy objects, but it overfits and requires more data to work well. Other CNNs for automatic polyp segmentation were found to be, such as ResUNet [12], which exploits the residual blocks to enrich the AP of polyp location, and HarDNet-DFUS [13], which merges HarDBlock with its homemade decoder Lawin Transformer, to improve the performance of accuracy and estimation speed. ResUNet can use powerful expression abilities but may need more data and computation to train. HarDNet-DFUS is real-time prediction-oriented and may trade off some accuracy. Colon Former [14] introduces attention and has a refined module with attention to produce finer output with a classical U-Net decoding structure. Attention-based methods could work well for large or complicated input images; however, they are computationally demanding and optimizing them can be difficult. MSRF-Net [15] is a CNN-based architecture particularly created for medical image segmentation. It is based on a DSDF (dual-scale dense fusion) block aggregating features of multiple scales and different receptive fields to maintain resolution and facilitate information propagation/flow. However, this method may not work well on images with too low contrast.

### 2.2. Transformer

As the methods mentioned above have attained good performance for automatic polyp segmentation, other techniques that utilize transformers are especially suitable for this task. These methods generally employ a pre-trained vision transformer (a type of encoder pre-trained on a relatively large dataset, e.g., ImageNet [16]) to obtain the feature information from the input image, for example, an adaptable vision transformer representation. Instead, these features are passed to a decoder that operates on the multi-scale features and merges them to obtain a final output. Such methods include FCN-Transformer [17] and SSFormer-L [18], which are demonstrated to outperform the existing SOTA on the Kvasir Segmentation Dataset.

**Figure 3:**
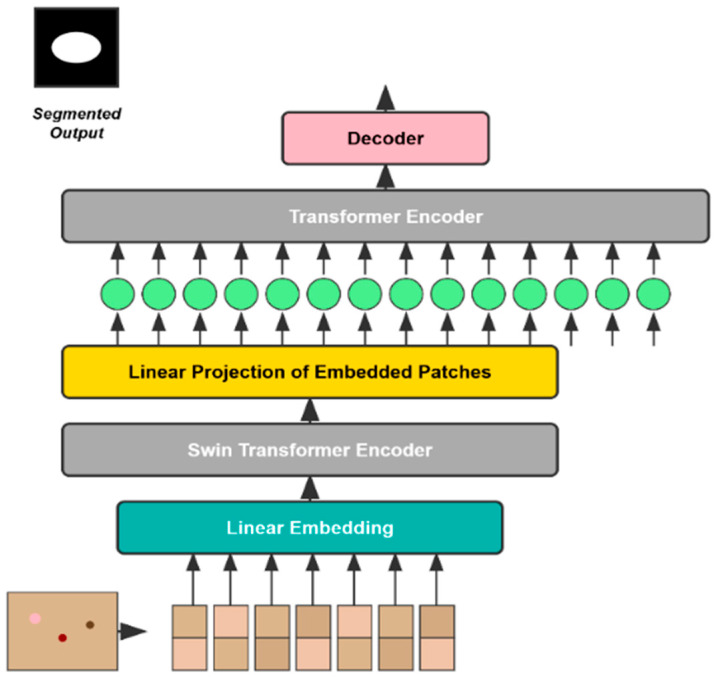
Transformer Architecture on performance for automatic polyp segmentation.

Transformers have recently become powerfully attractive in the computer vision (CV) community, largely thanks to their successful applications in natural language processing (NLP) and great performance in maintaining global content contexts. Vision-transformers (ViT) [19] mimic NLP as they capture the global content by the attention mechanism [20] that takes into account the entire large image patch to obtain the relevant information. Even though ViTs [19] work well in the CV setting, they are surpassed by traditional CNN models, such as EfficientNetV2 [21]. In all widely used image classification datasets (ImageNet [16] or CIFAR-10 [22]), this shows that we can still design more efficient CNN models. In Figure 3, an architecture for automatic polyp segmentation is shown.

Therefore, we investigate what traditional CNNs offer relative to the ViT architecture and how they can improve accuracy metrics. In general, this is an ongoing area of research, and different alternatives are under consideration. Thus, there is a requirement for additional study to identify the optimal model architecture and training approach. Trade-offs between accuracy, computational efficiency, and other performance measures must be carefully balanced when choosing a method for an application. HRENet [23] designed an informative context enhancement module to focus on hard regions. Nguyen et al. [24], Liu et al. [25], and Kim et al. [26] used the combination of reverse attention and boundary information to obtain the fine-grained boundary cues of polyps. Other methods also explored other aspects MSNet [27] presented a multi-scale subtraction network that eliminated redundant information and enhanced the complementarity of multi-level features. Guo et al. [28] used a mixup-based data augmentation strategy to learn more robust segmentation. MSRF-Net (Srivastava et al. [29]) utilized the Dual-Scale Dense Fusion (DSDF) block to fuse and exchange multi-scale features in different receptive fields and shape stream network to enhance polyp boundary. Polyp-PVT [30] adopted a transformer-based encoder to replace the traditional CNN backbones. FANet (Tomar et al., 2022 [31]) leveraged information from past training epochs to refine predictions in subsequent epochs, and TGANet (Tomar et al., 2022) [31] introduced an auxiliary text-weighted classification task to facilitate feature representation learning. However, these methods still could not handle the color inconsistency of polyps effectively, or the network could only achieve partial boundary localization.

Recent progress in cancer research has put a greater focus on the integration of multi-source heterogeneous data, such as genomics, imaging, and electronic health records (EHRs). Foundation models (FMs), a new class of pretrained deep-learning models with massive scale, present novel opportunities to biomarker discovery, cancer diagnosis and risk assessment, and personalized treatment. As surveyed in [32], multimodal integration strategies, computational frameworks, and public benchmarks/data resources have propelled a transition from machine learning (ML) to FMs in oncology and are laying the groundwork for the next generation of large-scale AI models and methods for data-driven discovery and translation in cancer.

## 3. Methodology

This section describes the methods and materials we used to accomplish our purpose. We implemented our proposed pipeline using various tools and techniques, including model deployment and model block explainability.

### 3.1. Proposed Segmentation Pipeline

**Figure 4:**
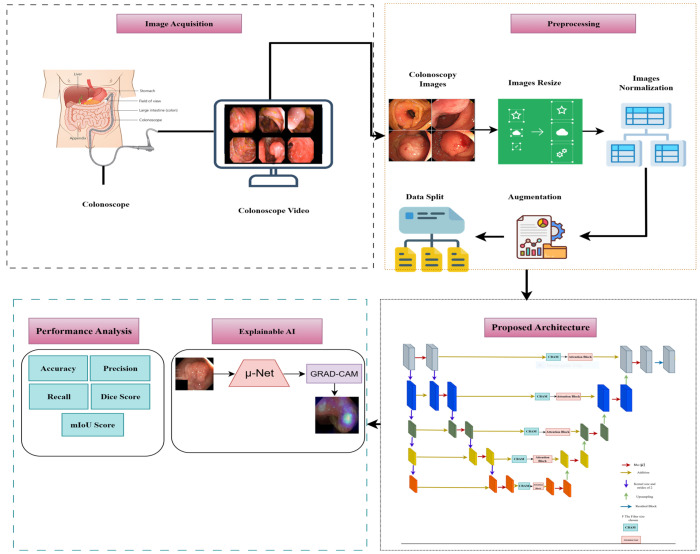
Our proposed research workflow.

The proposed µ-Net with CBAM is a sophisticated model specialized in binary segmentation of medical images, such as polyp segmentation. It follows an encoder–decoder configuration to retain high-level and low-level features for the best segmentation accuracy. The architecture comprises two new components: the µ-NETv2 block and CBAM. The µ-NETv2 block runs in parallel with six convolutional processes, and the network can train and determine the most useful features, which might degrade some details in the layers after it. Our proposed model attaches a second downscaling U-Net layer, not applied to the image, where some critical information is maintained to retain the low-level features. There are five Conv2D layers for the down-sampling path, and the encoders employ a µ-NETv2 block modified with CBAM for spatial and channel-wise attention. The bottleneck processes high-dimensional features to enhance the representation capacity of the network. In the upsampling path, features are progressively up-sampled with nearest neighbor interpolation and updated through skip connections to be enhanced by the µ-NETv2 blocks. The result is a binary segmentation mask created using a Conv2D with a Sigmoid activation function. The CBAM modules embedded in enable dynamic feature maps to be recalibrated to improve segmentation accuracy. Architecture retains spatial and contextual information, increases the keenness of boundaries, and deepens nets with a residual connection, thus contributing to better segmentation accuracy in medical imagery. Figure 4 shows our full workflow, which follows specific steps to complete the process.

### 3.2. Model Architecture

**Figure 5:**
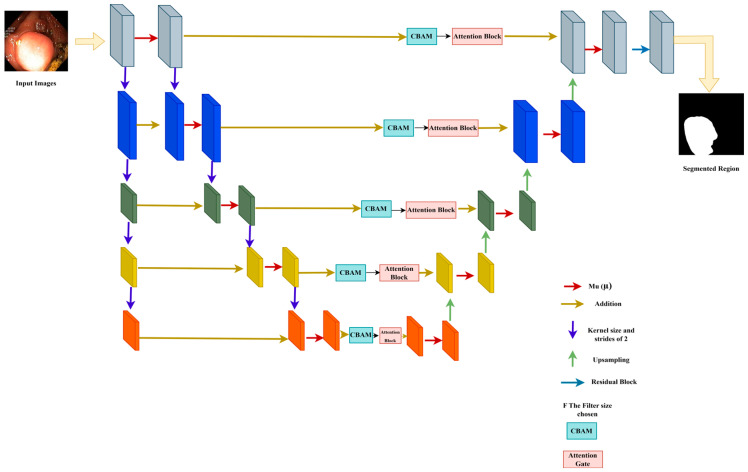
Architecture of the proposed study model (µ-Net).

The µ-Net with CBAM incorporation structural model is tailored for polyp segmentation based on medical images (e.g., colonoscopy or CT). This model adopts a popular encoder–decoder architecture (Figure 5), which can help extract and recover spatially dependent information. The encoder down-samples the spatial dimensions of the input image while selecting relevant features, and the decoder then up-samples the feature maps to the original resolution and generates the segmentation output.

In the encoder branch, the image is down-sampled iteratively with the convolutional layers (Conv2D), and the spatial resolution of the feature is reduced while learning significant information. The µ-block is the most important element in this path, where six parallel tensor convolution operations are employed for the adaptive selection of the most informative features from the input tensor in the early training stage [12]. These blocks concentrate on the most important details, like polyps, and discard some minor details, which the decoder takes care of. The kernel size of 2 with strides of 2 down-samples the image effectively to capture abstract features essential for identifying polyps. CBAM modules are directly incorporated within the µ blocks and provide spatial and channel attention. The spatial attention mechanism identifies sensitive and important regions that are dominated by areas that can contain polyps. Meanwhile, channel-specific attention highlights informative feature channels and focuses on critical features for polyp detection. Attention blocks polish the feature map through reweighting, which also improves the segmentation results of the model.

The bottleneck is the innermost part of the network, where the spatial resolution of the feature maps is reduced to its minimum. In this context, the network performs important high-level feature extraction and abstraction. The skip connections in the residual blocks help to retain important information learned at shallower layers and protect the model from forgetting significant features with increasing depth. Those residual blocks, equipped with CBAM, contribute to HSFA by refining the feature maps and optimizing their information on the model’s details of interest. The feature maps are then upsampled to the original image resolution decoder path. The above encoding process can lead to the loss of detailed information when segmenting polyps. To address this problem, adopting the nearest-neighbor interpolation in the up-sampling layer helps restore the spatial resolution to keep the fine details, irrespective of the object size. These skip connections combine low-level features of the encoder with the up-sampled features, allowing important information, e.g., edges and textures, to be preserved. After every upsampling step, the features go through another E block, regenerating the feature map that is reconstructed with high quality. The last layer is a 1 × 1 Conv2D with sigmoid activation to produce the final output. This layer’s output is a binary segmentation mask in which each pixel denotes the probability of belonging to the polyp. Using a sigmoid function, the resulting output is a probability map of polyps vs. background. Last, using CBAM blocks in both the encoder, and the decoder helps improve the performance by recalibrating the spatial and channel-wise features. Since these feature maps are informative, they work as an attention mechanism to assist the network in concentrating on polyps and enhancing important features that deserve to be picked out for segmentation. This procedure of dynamic recalibration facilitates the model’s work more effectively by paying more attention to the critical region and sharpening the segmentation boundary for polyp detection.

### 3.3. Block Components

#### 3.3.1. CBRes Block

**Figure 6:**
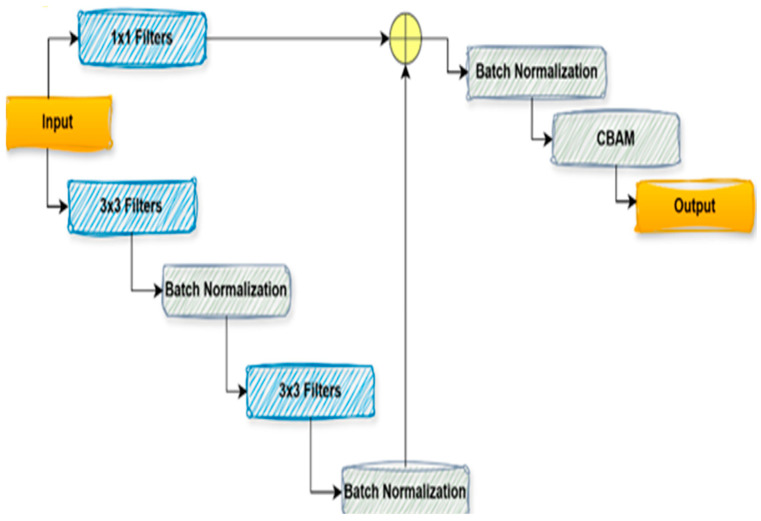
CBRes Block of proposed µ-Net.

Figure 6 represents an architectural component to improve deep neural networks’ efficiency and learning capability. A CBRes block is created using several terms. It starts with the input feature map and reduces the channel number or lowers the spatial information by using 1 × 1 convolutional filters, resulting in improved computational cost of the network. The feature map is subsequently fed through two 3 × 3 convolutional filter pairs, which learn spatial patterns in the image. Batch Normalization follows each convolution to stabilize learning by normalizing the activations to a fixed distribution, enabling the network to converge more quickly and be less sensitive to hyperparameters. After the convolutions, the feature map is refined with a CBAM (Convolutional Block Attention Module) block, which includes spatial and channel attention mechanisms. The attention modules concentrate the network’s attention on the informative features, improving the quality of the feature map by re-scaling important regions and channels. Finally, a residual connection is added to the original input and the input of the block to let the network easily learn the deep representation without facing the vanishing gradient problem. In this way, learning efficiency is preserved, and the loss of information in the layers is avoided. At last, the processed feature map, which passes through the residual connection layer and the CBAM block, is delivered as the output to the next layer, and the feature map’s high and low trajectory features are well maintained. The transformation performed by the proposed CBRes block can be mathematically expressed as follows:

(1) $$Y=CBAM(BN\left(F_{1\times 1}\left(X\right)+BN\left(F_{3\times 3}^{\left(2\right)}\;\left(BN\left(F_{3\times 3}^{\left(1\right)}\left(BN\left(X\right)\right)\right)\right)\right)\right)$$

$F_{1\times 1}^{\left(0\right)}$, $F_{3\times 3}^{\left(1\right)}$, and $F_{3\times 3}^{\left(2\right)}$ denote convolutional layers with respective kernel sizes. Batch Normalization (BN) is applied after each convolution, and CBAM is used to refine the features at the end.

#### 3.3.2. Dual Dilate-CB Block

**Figure 7:**
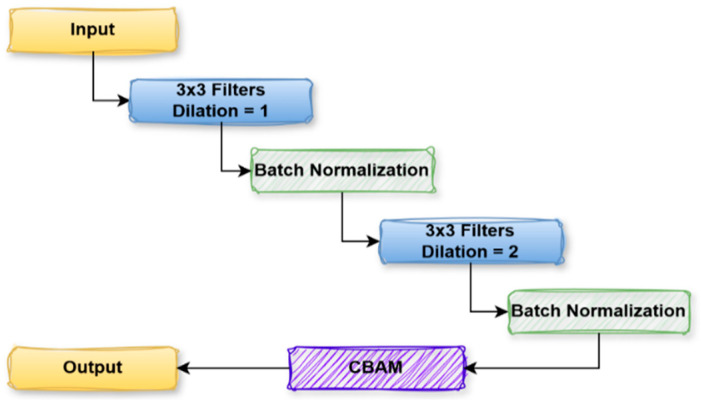
Dual Dilate-CB Block of proposed µ-Net.

In Figure 7, the DualDilate-CB block is an important module in independent learning models that wants both local feature extraction and a bigger receptive field of attention. It derives from the input feature map and applies a 3 × 3 convolutional filter with a dilation rate of 1 to the captured images’ local and spatial features. Then, Batch Normalization normalizes the feature map and stabilizes the training. The resulting feature map then goes through another 3 × 3 convolutional filter with a dilation rate of 2 to enlarge the field of view, which allows the model to learn more distant context features. After this transformation, Batch Normalization is reapplied. The processed feature maps are further fed through a CBAM, which recalibrates the features with attention at spatial and channel-wise levels. The spatial attention indicates the key areas of the image, and channel-wise attention denotes the critical feature channels, which help the model focus on the relevant parts of the image. Last, the output feature map is fed to the next network layer. This technique is useful in models where knowledge of local details and larger context patterns is important, as is the case, e.g., in segmentation or object detection. The dilated convolutional attention block transforms the input X with the two successive dilated convolutions with increasing dilation (actually, it is 1 and 2, respectively) and the two batch normalizations (BN), and the output is polished by CBAM as defined in Equation (2).

(2) $$Y=CBAM(BN\left(\left(F_{3\times 3}^{\left(d=2\right)}\;\left(BN\left(F_{3\times 3}^{\left(d=1\right)}\left(X\right)\right)\right)\right)\right)$$

#### 3.3.3. TriDilation Attention Block

**Figure 8:**
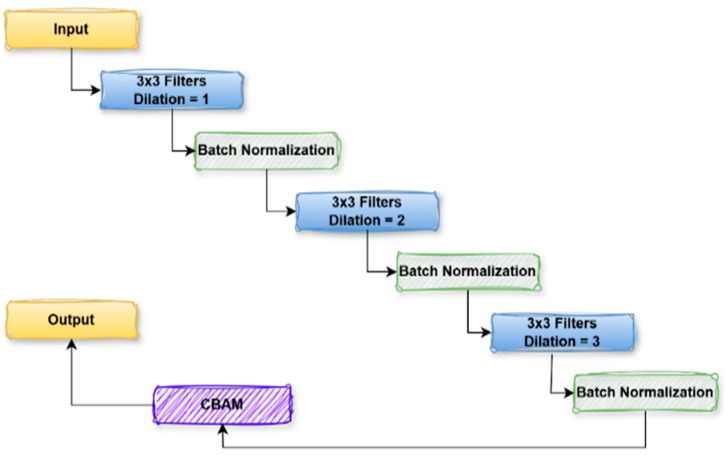
µ-Net TDAU Block.

The TriDilation Attention (TDAU) Block in Figure 8 is also a basic unit in the deep learning algorithms to handle local-global features by gradually expanding the receptive field’s size. It begins with the input feature map initially convolved with 3 × 3 kernels at a dilation rate of 1 to capture fine-grained spatial structures. Subsequently, we apply Batch Normalization to stabilize the activations. The subsequent feature map is then convoluted by another 3 × 3 convolution with a dilation rate of 2, which is used to capture large and more global patterns in the images, followed by Batch Normalization to retain the training stability. This BN is followed by a third 3 × 3 convolution with a dilation rate of 3 to the feature map, to increase the receptive field further to perceive long-range features.

After this block, batch normalization is applied again before the feature map is flowed through the CBAM, which promotes the spatial and channel-wise attention mechanism. The spatial attention pays more attention to the informative regions of the input image, and the channel-wise attention concentrates more on the significant feature channels. The output of its feature map is the refined features, fed into the subsequent layer of the network. This architecture fuses local and global feature extraction with attention mechanisms and thus applies to cases in which the network must learn complex patterns at various scales. The CBAM-enhanced dilated convolutional block comprises a sequence of three 3 × 3 convolutions with increasing dilation rates (1, 2, and 3) followed by batch normalization. A CBAM module also refines the last output in Equation (3).

(3) $$Y=CBAM(BN\left(\left(F_{3\times 3}^{\left(d=3\right)}\;\left(BN\left(F_{3\times 3}^{\left(d=2\right)}(BN(F_{3\times 3}^{\left(d=1\right)}\left(X\right)\right)\right)\right)\right)$$

#### 3.3.4. SplitFusion Block

**Figure 9:**
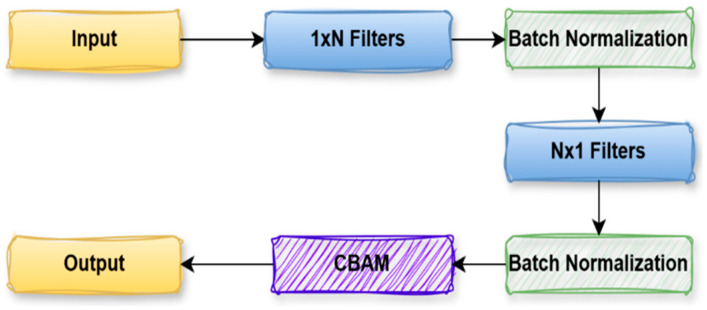
Split Fusion Block.

Figure 9 shows the SplitFusion Block, a generic module engineered to maximize feature representation for polyp segmentation. The process starts from an input feature map, which is first filtered with a set of 1 × N convolutional filters. This operation collects spatial salient features (horizontally), and Batch Normalization normalizes the outputs to reduce the training complexity. The normalized features are then processed by N × 1 convolutional filters to extract vertical features, followed by another Batch Normalization layer. This cascade asymmetrical convolution enables the network to extract directional features more effectively than using a square kernel as the standard. Following these convolutions, the output is sent through a CBAM, which adaptively enhances or suppresses the feature map by focusing on discriminative regions and ignoring the background. Lastly, the refined output is fed forward, strengthening the network to be more capable of precisely segmenting polyps through highlighting significant spatial and channel-wise features. The separable convolution block consists of a 1 × N convolution followed by an N × 1 convolution with batch normalization after each layer. Afterwards, the obtained feature map is further calibrated via CBAM Equation (4).

(4) $$Y=CBAM(BN_{2}\left(F_{N\times 1}\left(B_{N1}\left(F_{1\times N}\left(X\right)\right)\right)\right)$$

#### 3.3.5. µ(mu) Block

**Figure 10:**
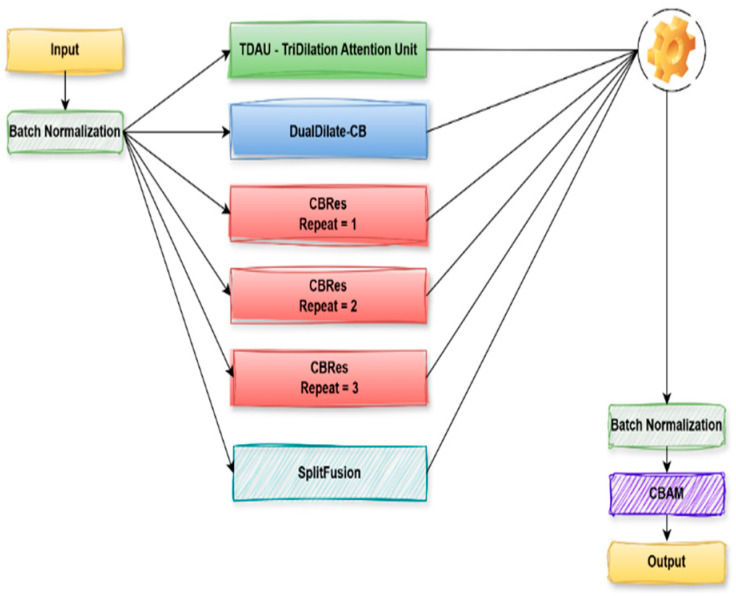
μ(mu) Block.

The architecture of the μ(mu) Block, a dedicated feature extraction module for polyp segmentation, is depicted in Figure 10. The input is first batch-normalized to ensure stable feature distributions. It is then followed by processing the normalized input through multiple parallel paths so that diverse and complementary information may be well captured. These branches are a TriDilation Attention Unit (TDAU) for multi-scale contextual attention, a DualDilate-CB block to multi-extend receptive field expression, and three cascaded CBR (Convolution-BatchNorm-ReLU) residual blocks (CBRes) to achieve the feature representation with increased depth. Furthermore, a SplitFusion process combines spatially non-local characteristics. In the last column, output from all of these branches are concatenated or merged at a single operation node. The concatenated output is batch-normalized and further processed by a CBAM (Convolutional Block Attention Module) that uses spatial and channel-wise attentions to enhance informative features and dampen noise. Finally, the refined output is passed to the latter parts of the segmentation network. This form of rich and varied processing throughout the μ (mu) block greatly enhances the ability of the network to identify and outline polyps accurately. The μ-block concatenates the six parallel feature branches with the fusion operator and then refines them using Batch Normalization and CBAM as described in Equation (5).

(5) $$Y=CBAM(BN(F_{fuse}(\sum_{i=1}^{6}\beta_{i}\left(X\right))))$$

where $\beta_{i}\left(X\right)$ is each of the six parallel branches, F_fuse_ is a Feature fusion operator, and BN is Batch normalization.

### 3.4. Model Evaluation

For polyp segmentation, researchers used classic performance evaluation metrics for image segmentation, such as Dice Coefficient (DSC), Intersection over Union (IoU), Precision (P), Recall (R), Average Precision (AP), and pixel accuracy. The next section outlines the various evaluation metrics through which our methods’ performance has been assessed for polyp segmentation.

(1) Dice coefficient:

The DSC [33] is a widely used metric for comparing pixel-by-pixel prediction with the ground-truth. It is described as:

(6) $$DSC\;\left(A,B\right)=\frac{2\;\times \left|A\cap B\right|}{\left|A\right|+\left|B\right|}\;=\;\frac{2\;\times TP}{\left(2\times TP\right)+FP+FN}$$

(2) Intersection over union:

The intersection-over-union (IoU) is commonly used in polyp segmentation. It is calculated by counting the overlapped pixels between the target and predicted masks and dividing them by the total number of pixels for both. It ranges from 0 to 1, where 0 means no overlapping and 1 represents complete overlap. The mean IoU is obtained by averaging the IoU for a binary (two classes) or multi-class segmentation. There is a standard method [34].

(7) $$IoU\left(A,B\right)=\frac{A\cap B}{A\cup B}=\frac{TP}{TP+FP+FN}$$

(3) Pixel accuracy:

Accuracy is a measure of a model’s performance over the classes. It is handy if all classes count. The percentage of accurate predictions is calculated by unwinding the actual number of correct predictions by the overall number of forecasts, Source [34]. Another way to assess the performance of image segmentation algorithms is to measure the proportion of pixels on a given image that were imaged or classified correctly. All-class pixel accuracy is commonly reported per class and averaged across classes. A binary mask is created to compute the per-class pixel accuracy. Positive (negatively) pixels labeled are called real positive (negative), whether or not they are predicted as positive.

(8) $$Pixel\;Accuracy=\frac{TP+TN}{TP+TN+FP+FN}$$

(4) Precision:

The number of boundary pixels in the automatic segmentations that coincided with those in the ground truth. Precision is calculated as the number of Positive image samples that are correctly classified divided by the total number of them classified as Positive [35].

(9) $$Precision=\frac{TP}{TP+FP}$$

(5) Recall:

Recall is the proportion of the ground truth boundary pixels accurately detected by the automatic segmentation. The fraction of the Positive samples in images is classified correctly as Positive. It measures how well the model can find the positive samples. The higher the recall, the more positive samples are detected [35].

(10) $$Recall=\frac{TP}{TP+FN}$$

## 4. Experimental Results

### 4.1. Implementation Details

We compared the considered models using the same training, validation, and test set to allow direct and fair comparisons between each model. In detail, the splits of each dataset are conducted randomly with the ratio of 80:10:10, for training, validation, and testing. This split was made to ensure that the decision was unbiased and that the model comparison was fair. We present the split datasets in the section “Data Availability” to enable the reproduction of our results. We designed our experimental setup to validate the state-of-the-art performance of our and competing models on hold-out data and demonstrate our model’s capability to generalize to highly varied settings. In the former case, we evaluated the datasets as independent tasks and compared the model’s performance with the other methods. That way, we could logically quantify its generalization and predictability when testing new observations. This cross-dataset validation indicated a strong generalization of our model on different datasets without extra pre-training data. We train our model to predict binary segmentation maps for RGB images. The input images are resized to 352 × 352 pixels to reduce computation complexity. We used a Lanczos 30 filter to guarantee the quality and avoid aliasing when resizing the images. We optimize AdamW with a learning rate of 0.0001 and a weight_decay of 1 × 10^−5^. The models were trained for 300 epochs with a batch size of 2. The architecture was realized using Tensorflow 2.11, and training was conducted on an NVIDIA GeForce RTX 3060 (NVIDIA Corporation, Santa Clara, CA, USA).

To obtain the best training stability and performance, we empirically set all hyper-parameters by using a grid search. The learning rate, batch size, and the number of epochs were varied within $\left[5\times 10^{-5},\;5\times 10^{-4}\right],\left[1,\;4\right]$ and set to [100, 400], respectively, and we use the hyper-parameter combination with the best validation Dice and the least validation loss as our final hyper-parameter (learning rate = 1 × 10^−4^, epochs = 300, batch size = 2). For a balance between retaining the subtle polyp boundaries and the computational efficiency and fair comparison with other state-of-the-art networks, the resolution of 352 × 352 for the input image was used, where a lower resolution (e.g., 256 × 256) would lead to a loss of sharpness at the polyp boundaries, and a higher resolution (e.g., 512 × 512) would only bring an increased training time with slightly improved performance. We use Lanczos filter for resizing the input images, since it has a better edge-preserving and anti-aliasing than the bilinear or bicubic interpolation, which further helps retain the sharpness along the subtle polyp boundaries. The sensitivity analysis shows that μ-Net is stable to the learning-rate in the range of 5 × 10^−5^ to 5 × 10^−4^, and best converged around 300 epochs, after which a minor over-fitting is observed.

### 4.2. Dataset Overview

The Kvasir-SEG dataset [36] contains 1000 polyp samples (video frames) with corresponding ground truth segmentation masks. These are taken from the Kvasir Dataset v2, a popular GI medical image analysis dataset. The resolutions of the images range from 332 × 487 pixels to 1920 × 1072 pixels, which is within a broad spectrum suitable for a robust polyp detection model. The original photos and their corresponding segmentation masks are provided and organized into separate folders with matching filenames for convenience. Images are supplied in JPEG format for online viewing and downloading.

### 4.3. Structure and Annotation of the Dataset

A JSON file, along with the dataset, describes the bounding boxes of each polyp image. Such bounding boxes are important in recognizing the rough position of the polyps in the photos, which may be part of the preparation steps when training detection algorithms. Moreover, the corresponding segmentation masks give accurate pixel-level annotations on the polyps, with high-quality ground truth for training and evaluating the segmentation model.

### 4.4. Dataset Augmentation

We treated the training set with data augmentation, and the model learned smoothly (high generalization ability), so regularization methods with dropout were unnecessary. The augmentation was accomplished using the Album Enations library [37]. This process captured random shifts that help generalize the image model to the unseen data. For each epoch, we augmented our training inputs using random transformations based on previous work, but modified them, considering the specific needs of our models. Data augmentation is a method to produce new data points or data elements by using the original dataset to expand the data size. This approach could enhance the performance of Deep Learning (DL) models by providing them with a more extensive collection of training examples. Of course, there are data gathering and labeling costs, but data augmentation reduces operational costs by enriching the dataset with it. Data augmentation techniques were performed on μ-Net’s training dataset to increase model robustness and generalization (Table 1). The geometric augmentations are: Horizontal Flip (probability of 0.5), Vertical Flip (probability of 0.5), Random Rotate 90° (probability of 0.3), Rotation (±30°), Translation (±10%), Scaling (90–120%), Shear (±10°), and Affine Transform (probability of 0.7). These transformations expose the model to input data with different orientations, positions, and distortions. Photometric augmentations are: ColorJitter (probability of 0.5), Brightness (0.6–1.6×), Contrast (±20%), Saturation (±10%), and Hue (±0.01). These augmentations make the model more robust to lighting conditions and color variations in the input data. All the data augmentation techniques are implemented with Albumentations library, and the seed for data generation is fixed for reproducibility.

### 4.5. Explainable AI

XAI is a class of techniques that provide insight into the decision-making mechanism of AI models for humans [38]. In contrast to the classical “black box” models, whose predictions are difficult to understand, the goal of XAI is to ensure that the AI systems of the future will explain their forecasts in a manner that is assertable and interpretable. This process is especially crucial in sectors such as health care, where AI systems are deployed to identify diseases or interpret medical images. In medical image segmentation, for example, XAI will help doctors understand why a model believes certain parts of an image are important, such as a tumor or polyp. These techniques, such as LIME, SHAP, and Grad-CAM, give us a post hoc explanation where we know how the model arrived at certain predictions, for example, which part of an image contributed the most towards the model making a prediction. XAI increases the trust and trustworthiness of AI systems by making them more transparent, responsible, and interpretable, which is crucial in domains where Precision and justification are essential.

**Figure 11:**
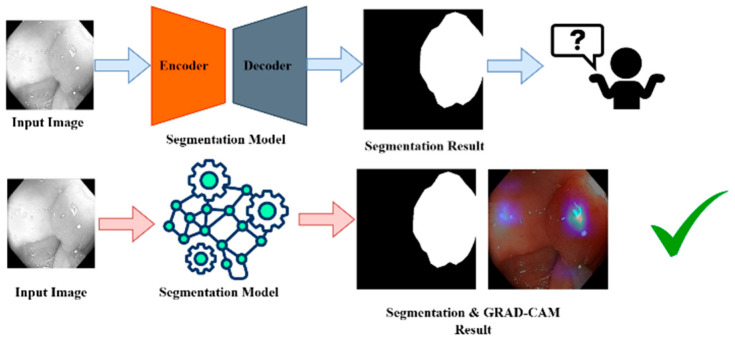
XIA framework for GRAD-CAM result analysis. “✓” means that grad-cam result become effective and trustable.

Nevertheless, the trade-off between the accuracy of complex models, including deep learning, and their explainability is an open problem since models with better Precision tend to be more difficult to explain. However, as part of their interoperability needs, they must also provide XAI so that AI systems become effective, comprehensible, and trustworthy. Figure 11 shows this for better understanding.

## 5. Results

### Ablation Study

We introduced the proposed model of μ-Net by tuning the hyperparameter values via grid search, as illustrated in Table 2. The network with the maximum Dice coefficient in validation was chosen for the proposed system.

To assess the impact of each component on our proposed µ-Net, we performed an ablation study against several U-Net variants. As reported in Table 3 , the baseline U-Net achieved a Dice score of 93.12% ± 0.45 with an IoU of 0.881. When the CBRes module was integrated, there was a slight decrease in the Dice score to 92.87% ± 0.52 and IoU to 0.862, suggesting that while the module might enhance some feature representations, it could also introduce additional complexity that slightly hinders performance. The variant incorporating the Dual Dilate-CB module saw further degradation in performance (Dice: 91.34% ± 0.60, IoU: 0.842). This result implies that multi-scale dilation alone is insufficient without effective feature integration. The TriDilation variant also underperformed with a Dice score of 90.75% ± 0.58 and IoU of 0.831, while the SplitFusion variant showed the lowest performance (Dice: 89.61% ± 0.63, IoU: 0.815), reflecting that naive feature fusion or dilation strategies are inadequate for capturing the complex polyp boundaries. In contrast, our proposed µ-Net demonstrated superior performance, achieving the highest Dice score of 94.02% ± 0.38 and IoU of 0.8772. It also achieved the lowest FLOPs (39.04 G) and latency (15 ms), with the *p*-value being consistently at the lowest level (0.001), ensuring the statistical significance of the improvement. These findings confirm that the proposed architectural improvements in µ-Net are effective in capturing fine-grained polyp features and maintaining computational efficiency, making it a highly suitable solution for real-time polyp segmentation.

**Table 2:** Proposed model input parameters.

Parameter	Value
Img_size	352
Dataset_type	Kvasir
Filters	17
Seed_value	42
Learning_rate	1 × 10^−4^
Epochs	300
Min_loss_for_saving	Np.inf

**Table 3:** Ablation study results for polyp segmentation comparing baseline **µ**-Net and its variants.

Variant	Dice (%) ± std	IoU	FLOPs (G)	Latency (ms)	*p*-Value
U-Net (Baseline)	93.12 ± 0.45	0.881	48.7	22	0.010
U-Net + CBRes	92.87 ± 0.52	0.862	47.5	21	0.012
U-Net + Dual Dilate-CB	91.34 ± 0.60	0.842	45.2	20	0.008
U-Net + TriDilation	90.75 ± 0.58	0.831	44.8	20	0.004
U-Net + SplitFusion	89.61 ± 0.63	0.815	43.5	19	0.003
Proposed µ-Net	94.02 ± 0.38	87.72	39.04	15	0.001

**Figure 12:**
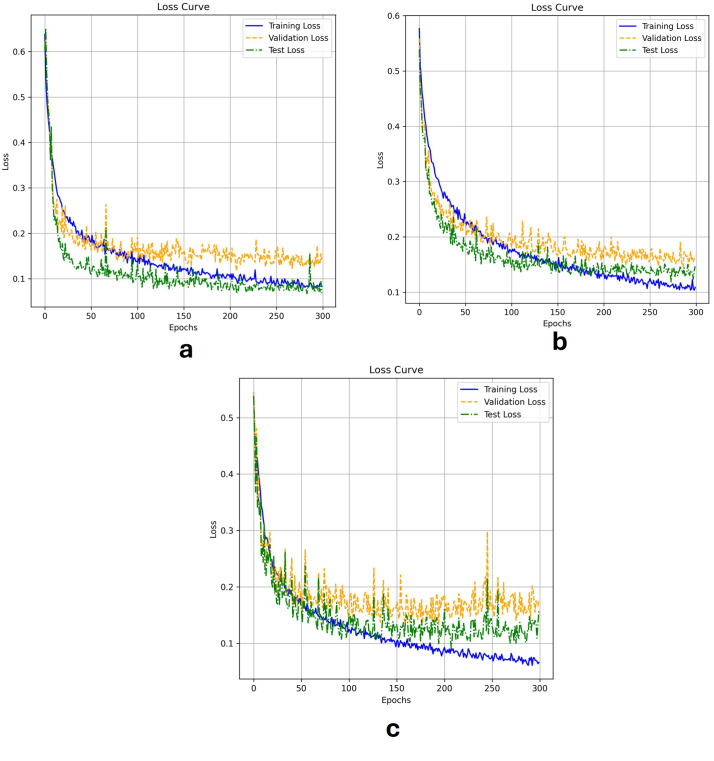
Loss Curves for (**a**) μ-Net, (**b**) Attention U-Net, and (**c**) ResUNet++ Models During Training, Validation, and Testing.

Figure 12 shows Loss Curves for Segmentation Models (μ-Net, Attention U-Net, and ResUNet++) representing their training, testing, and validation loss for 300 epochs. The training loss curves descend smoothly for the ResUNet++ (Figure 12c), suggesting good learning; however, the validation loss curve fluctuates in the latter part, indicating possible over-fitting. The test loss has roughly the same shape as the validation loss but slightly deviates at later epochs, indicating that the model might not generalize as well as we would like. In the Ablation study, the attention U-Net has robust and less erratic training dynamics, where the training loss decreases more smoothly compared to ResUNet++. The validation loss is more stable, which means attention brings better overfitting prevention to our model.

In contrast, μ-Net (Figure 12a) demonstrates the most desired results as its training loss presents much less erratic and even decreases, resulting in more effective learning. The variance of μ-Net on validation loss is relatively lower than that of the other two models, implying that μ-Net has higher stability. The test loss of μ-Net is the least among the three models, indicating that it has the best generalization ability, and the reason can be attributed to the fact that it uses residual connections and dense skip pathways. Superior to ResUNet++ and Attention U-Net, μ-Net achieves the best generalization performance and training efficiency, making it the most reliable model for medical image segmentation tasks such as polyp detection.

**Figure 13:**
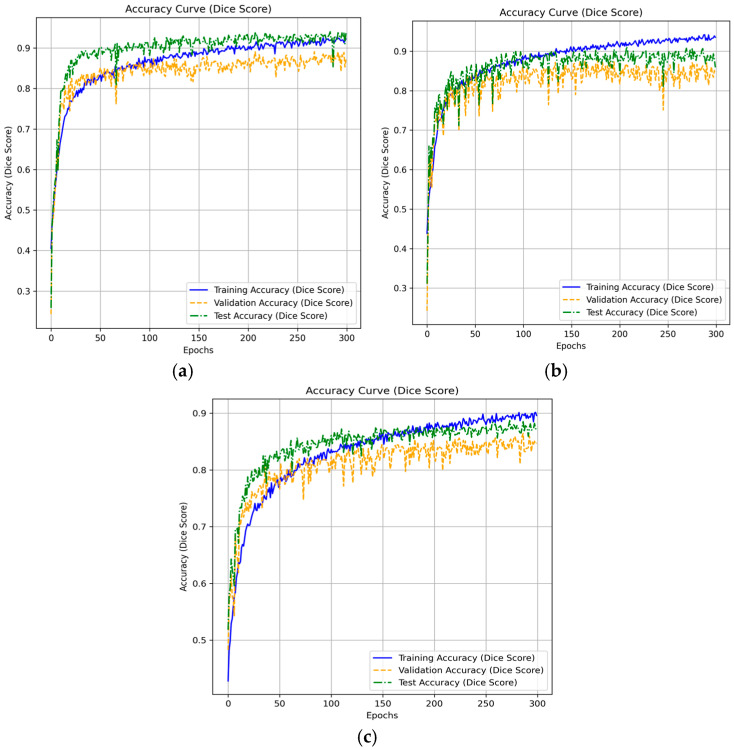
Dice Score for (**a**) μ-Net, (**b**) Attention U-Net, and (**c**) ResUNet++ Models During Training, Validation, and Testing.

Figure 13 gives the results of the ResUNet++ model (Figure 13c). As the training accuracy continues to rise gradually, there is a noticeable dispersion between the validation and test accuracies after a few epochs. This dispersion indicates that the model learns the training data well, yet fails to generalize properly to new data. From the Attention U-Net (Figure 13b), one can see the convergence of training, validation, and test curves, which clearly follow the same shape as the two preceding cases; there is a clear matter of certainty throughout training. This curve shows that the Attention U-Net generalizes better than ResUNet++ to the validation and test sets, leading to a lower extent of overfitting. However, the fleeting perturbation of the validation and test curves suggests that it sometimes undergoes a non-stable learning. The μ-Net model suggests the most reliable and harmonized results among the three. The training, validation, and test accuracy converge well, indicating μ-Net not only learns a good representation of the training data but also generalizes well on both validation and test sets.

We also included SD estimations as part of our analysis to increase our model performance evaluation. The measure also offers insight into the variability of the metrics used between models and provides information on the performance of these methods. This statistical approach provides a valuable supplement to raw performance numbers, indicating how consistently and reliably the model has performed. To make a fair comparison, we applied image augmentations for the base U-Net [7] model in our previous publication on the same dataset. These augmentations were the same as those used in our model. Furthermore, we also indicate which models were pre-trained in the tables to shed light on the results. Quantitative comparison with baseline methods in terms of segmentation performance is illustrated in Table 4. Our proposed μ-Net achieves the best performance against all baseline methods on most evaluation metrics. It outperforms others in terms of Dice score (94.02%), Jaccard index (88.72%), precision (94.75%), and recall (93.31%). This suggests that our method offers superior overlap with the ground truth masks and is more robust in accurately delineating the target regions with fewer false negatives. Despite incorporating multi-level attention and feature refinement modules, the proposed μ-Net achieves a relatively moderate number of parameters (39.18M) and GFLOPs (39.04), outperforming even the lighter models like Polyp-PVT in both accuracy and efficiency. Moreover, our model stands out with the fastest inference time (15 ms), reinforcing its potential for real-time clinical applications. In comparison, models such as Attention U-Net and Polyp-PVT demonstrated relatively lower segmentation accuracy and higher latency, further underscoring the effectiveness of the proposed architectural innovations.

**Figure 14:**
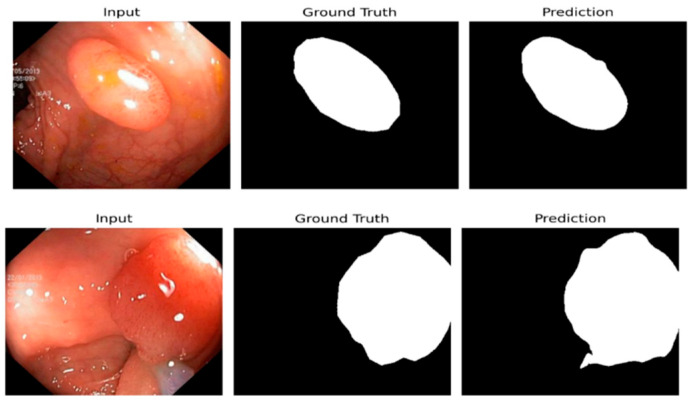
Predicted polyp masks.

The results of medical image segmentation are illustrated for input images, ground truth, and model predictions in Figure 14. This figure shows the input images, which are cross-section images from, for example, a colonoscopy, displaying internal body details. The Ground Truth includes human-annotated segmentation masks, where the areas of interest are marked in white, while those not in the areas of interest leave the rest of the part as black. The prediction shows the segmentation result of the model and indicates the predicted regions of interest. This figure generally facilitates the visual comparison of the model’s ability to detect and segment useful areas of medical images.

**Figure 15:**
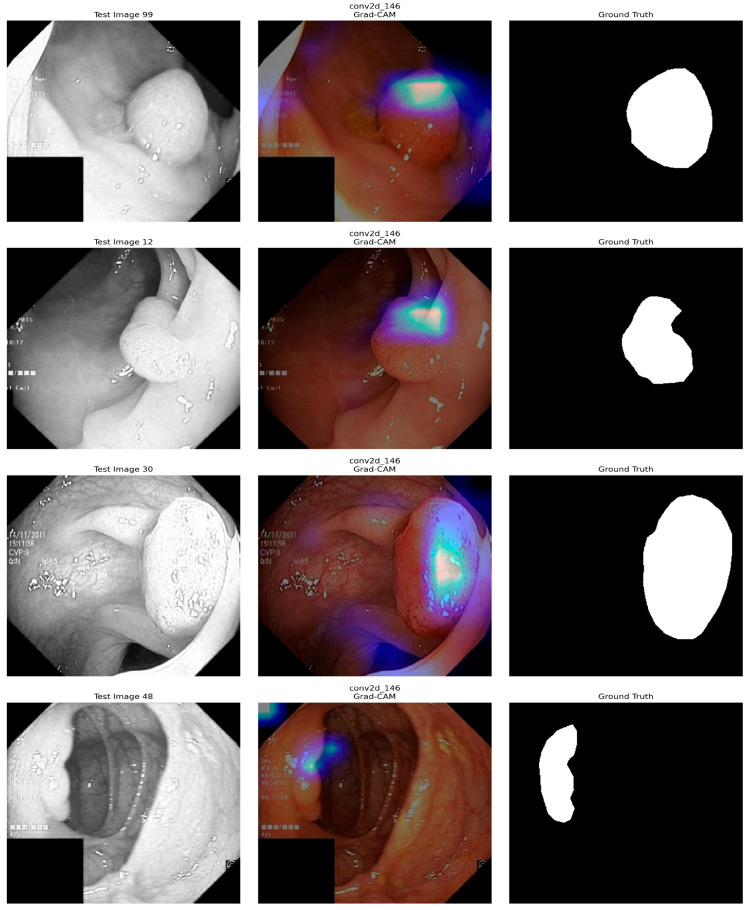
XAI (Grad-CAM) Analysis of our model.

Figure 15 shows the application of Grad-CAM to visualize which part of the input images the deep learning model is based on its predictions. The first sample images represent the test images, which are images of a medical nature, such as a colonoscopy. The second test image shows the Grad-CAM heatmaps, in which the colored areas indicate where the model has been focusing on when making predictions, and the color intensity stands for focusing strength. The last images correspond to the Ground Truth masks, the manually drawn regions of interest. Typically, abnormal areas are shown in white on a black background. By contrasting the Grad-CAM results with Ground Truth, one can determine whether model attention is consistent with the true regions of interest and assess interpretability and quality. This analysis helps to determine whether the model properly attends to the relevant areas to make an accurate prediction.

## 6. Discussion

The proposed system is an automatic polyp segmentation approach based on a deep learning model to identify polyps in colonoscopy checks automatically. It is intended to help diagnose and treat colorectal disease by identifying potential high-risk polyps early. Extensive experiments demonstrate that the proposed method outperforms other state-of-the-art methods regarding segmentation accuracy. The model has been highly accurate when tested on unseen polyp datasets derived from multiple imaging devices and laboratories. The segmentation approach is trained on the Kvasir-SEG dataset, which has achieved 98.01% accuracy. Furthermore, we also employed Grad-CAM technology, which provides explanations on the rationale and attention regions contributing to the decision of deep learning models, to demonstrate the proposed model’s generalization ability. Our system still demonstrates impressive stability and performance by training on heterogeneous datasets. To improve robustness, the system includes several processes, such as black pixel counting, image quality analysis, and color conversion, as pre-evaluation procedures. Although μ-Net performs well in polyp segmentation, there are some limitations. First, the model’s generalization could be restricted when facing different real-world clinical data because the benchmark dataset had been trained without considering differences in image quality, lighting, and patient demography. Furthermore, the detected performance of polyps with hazy or indistinct borders can be difficult. The computational complexity of μ-Net, concerning multiple convolutional blocks and residual layers, may also limit its application in resource-limited settings or real-time scenarios such as live colonoscopy. Additionally, although μ-Net generalizes satisfactorily in new subjects with limited training data, it can be potentially improved by employing larger and more diverse datasets with additional pre-training. Lastly, although our model performs well without pre-training, a comparison with alternative methods such as FCN-Transformer reveals the usefulness of pre-training for improving generalization across tasks, particularly when limited training data is available. These limitations indicate the potential for additional enhancements to μ-Net to optimize it for clinical use, particularly for processing complex, low-contrast polyps and for the response time required by real-time applications.

## 7. Conclusions

The proposed µ-Net paradigm presented in this paper can outperform the state-of-the-art in the problem of polyp segmentation in images taken by colonoscopy, either public or private data. The proposed method can effectively handle image information in multiple resolutions by taking advantage of the encoder–decoder model with the residual down-sampling block and the kernel convolution block. Meanwhile, the data augmentation strategy has also contributed to the model’s generalization. The µ-framework has shown better generalization ability and competitive results with limited training data. The µ-Net architecture can be used as an excellent candidate model in other segmentation tasks and needs further exploration.

## Figures and Tables

**Table 1:** The attributes are used in data augmentation techniques, and their values are.

SL	Matric	Value
01	HorizontalFlip	0.5
02	VerticalFlip	0.5
03	RandomRotate90	0.3
04	ColorJitter	0.5
05	Brightness	0.6 to 1.6×
06	Contrast	±20%
07	Saturation	±10%
08	Hue	±0.01
09	Affine Transform	0.7
10	Scale	90% to 120%
11	Translation	±10%
12	Rotation	±30°
13	Shear	±10°

**Table 4:** Segmentation performance and computational metrics of ResUNet++, Attention U-Net, Polyp-PVT, SANet and the proposed μ-Net on the Kvasir-SEG dataset.

Method	Dice (%)	Jaccard (%)	Precision (%)	Recall (%)	Params (M)	FLOPs (G)	Inference Time (ms)
ResUNet++	90.48	82.62	91.46	89.52	29.7	115.88	16
Attention U-Net	88.52	79.40	87.55	89.51	34.0	48.5	22
Polyp-PVT	84.60	77.60	85.50	89.50	37.0	41.58	19
SANet	90.80	85.40	89.30	91.17	27.7	44.40	21
μ-Net (Proposed)	94.02	88.72	94.75	93.31	39.18	39.04	15

## Data Availability

The Kvasir-SEG Data (Polyp segmentation & detection) dataset used for this study is publicly available at "https://www.kaggle.com/datasets/debeshjha1/kvasirseg" (accessed on 3 May 2025).
